# Evaluation of prospective motion correction of high-resolution 3D-T2-FLAIR acquisitions in epilepsy patients^☆^

**DOI:** 10.1016/j.neurad.2018.02.007

**Published:** 2018-10

**Authors:** Sjoerd B. Vos, Caroline Micallef, Frederik Barkhof, Andrea Hill, Gavin P. Winston, Sebastien Ourselin, John S. Duncan

**Affiliations:** a Translational Imaging Group, CMIC, University College London, London, United Kingdom; b Epilepsy Society MRI Unit, Chalfont St Peter, United Kingdom; c Wellcome/EPSRC Centre for Interventional and Surgical Sciences (WEISS), University College London, London, United Kingdom; d Neuroradiological Academic Unit, Department of Brain Repair and Rehabilitation, UCL Institute of Neurology, London, United Kingdom; e Department of Radiology and Nuclear Medicine, VU University Medical Center, Amsterdam, The Netherlands; f Department of Clinical and Experimental Epilepsy, UCL Institute of Neurology, London, United Kingdom; g Neuroimaging of Epilepsy Laboratory, Montreal Neurological Institute, McGill University, Montreal, Canada; h Dementia Research Centre, UCL Institute of Neurology, London, United Kingdom

**Keywords:** FLAIR, Prospective motion correction, Image quality, Epilepsy, FOV, Field-of-View, GM, Grey Matter, PMC, Prospective Motion Correction, PROMO, Prospective Motion correction (GE Healthcare proprietary term), SNR, Signal-to-Noise Ratio, WM, White Matter

## Abstract

T2-FLAIR is the single most sensitive MRI contrast to detect lesions underlying focal epilepsies but 3D sequences used to obtain isotropic high-resolution images are susceptible to motion artefacts. Prospective motion correction (PMC) – demonstrated to improve 3D-T1 image quality in a pediatric population – was applied to high-resolution 3D-T2-FLAIR scans in adult epilepsy patients to evaluate its clinical benefit. Coronal 3D-T2-FLAIR scans were acquired with a 1 mm isotropic resolution on a 3 T MRI scanner. Two expert neuroradiologists reviewed 40 scans without PMC and 40 with navigator-based PMC. Visual assessment addressed six criteria of image quality (resolution, SNR, WM-GM contrast, intensity homogeneity, lesion conspicuity, diagnostic confidence) on a seven-point Likert scale (from non-diagnostic to outstanding). SNR was also objectively quantified within the white matter. PMC scans had near-identical scores on the criteria of image quality to non-PMC scans, with the notable exception that intensity homogeneity was generally worse. Using PMC, the percentage of scans with bad image quality was substantially lower than without PMC (3.25% vs. 12.5%) on the other five criteria. Quantitative SNR estimates revealed that PMC and non-PMC had no significant difference in SNR (*P* = 0.07). Application of prospective motion correction to 3D-T2-FLAIR sequences decreased the percentage of low-quality scans, reducing the number of scans that need to be repeated to obtain clinically useful data.

## Introduction

The primary purpose of MRI in individuals with epilepsy is to detect focal epileptogenic lesions. Dedicated MRI protocols have been demonstrated to be superior to standard MRI protocols [1], leading to guidelines including 3D-T1, T2, and T2-FLAIR imaging [2], [3], [4], [5], [6], with T2-FLAIR the single-most sensitive over all lesion types (85%) [3].

For optimal detection of lesions it is critical to have high-resolution images, viewed in multiple slice orientations [3]. Isotropic 3D acquisitions can be reformatted to allow this, and can provide improved SNR over 2D acquisitions [7], resulting in 3D-T2-FLAIR sequences providing improved lesion conspicuity [2], [8], and sensitivity and specificity for epileptogenic lesions [9]. Isotropic 3D scanning also helps in computer-assisted lesion detection, where morphometric analysis of 3D-T2-FLAIR scans helps to highlight covert focal cortical dysplasias: lesions that were not detected initially but confirmed upon retrospective visual inspection [10], [11].

One issue with 3D sequences in routine clinical practice, and 3D-T2-FLAIR in particular, is the sensitivity to subject motion. The long inversion time for optimal CSF suppression and long recovery time to ensure adequate T1 relaxation means that a 1 mm isotropic 3D-T2-FLAIR scan may take 6–9 minutes [12], during which subject motion may degrade image quality, affecting the whole volume. Image-based prospective motion correction (PMC) tracks the subject's head and adjusts the field-of-view (FOV) when the head is moved, thereby largely negating motion artefacts [13]. In clinical settings, PMC has been shown to improve image quality and reduce artefacts in 3D-T1 scans in a pediatric population [14], [15] but has not been evaluated outside this single specific application. In this work, we evaluate PMC for 3D-T2-FLAIR acquisitions in adult epilepsy patients, to investigate its clinical benefits to overall image quality and scan time efficiency.

## Materials and methods

### Subject population

All subjects included in this study were consecutive patients scanned as part of their routine clinical imaging workup within the Epilepsy Society MRI Unit. The study was considered a service evaluation using clinically acquired data by the NHNN and the Institute of Neurology Joint Research Ethics Committee.

Two separate study groups were defined: 3D-T2-FLAIR without PMC; and PMC 3D-T2-FLAIR. Group 1 (non-PMC) is the department's routine 3D-T2-FLAIR protocol. Group 2 (PMC) is the PMC. Each group consisted of 40 consecutively scanned subjects, based on a sample size calculation of an initial scan rejection ratio of 15% in non-PMC (current estimate in our centre) and a 5% rejection ratio in PMC scans (conservative estimate by manufacturer), with power (1−β) of 0.8 and type 1 error rate (α) of 0.05 yielding a lower bound of 38 scans. Each subject had only a single 3D-T2-FLAIR scan. All patients were scanned within a three-month period with no other modifications to the scanner.

Mean age of subjects was 40.2 years old (range 17–75y), with a total of 39 male and 41 female subjects.

### MRI acquisition

3D-T2-FLAIR scans were acquired on a 3 T GE MR750 scanner (GE Healthcare, Milwaukee, US) with a 32-channel head coil. A 3D fast spin echo sequence with variable flip-angle readout (CUBE) was used with acquisition parameters as detailed in Table 1. The FOV was oriented in an oblique coronal plane along the long axis of the hippocampus.

**Table 1:** Acquisition parameters for the non-PMC and PMC scans.

	Non-PMC	PMC
FOV (AP × IS × RL)	224 × 256 × 256 mm	224 × 256 × 256 mm
Acquisition matrix (AP × IS × RL)	224 × 256 × 256	224 × 256 × 256
Resolution (AP × IS × RL)	1 × 1 × 1 mm	1 × 1 × 1 mm
TE	137 ms	142 ms
TI	1882 ms	1870 ms
TR	6200 ms	6200 ms
ARC (AP × IS)	2 × 2	2 × 2
Echo Train Length	150	150
Total scan time	7 m17 s	7 m24 s

FOV: field of view; AP: anterior-posterior; IS: inferior-superior; RL: right-left; TE: echo time; TI: inversion time; TR: repetition time; ARC: autocalibrating reconstruction for cartesian imaging.

In the subject group scanned with prospective motion correction, this refers to the method implemented on GE scanners, called PROMO [13], and is an image-based method to track head motion using three perpendicular 2D spiral navigators that are acquired multiple times between the end of the readout and the inversion pulse for the next excitation. These navigator images are used instantaneously to detect rigid-body head motion and to reorient the FOV accordingly. Additionally, any corrupted segments of k-space are reacquired at the end of the sequence. If many corrupted k-space segments need reacquiring, the maximum time allotted for this is 180 seconds. Integrating the navigators changes the TI and TE of the sequence and the total scan time, as detailed in Table 1. Similar to the original FLAIR protocol, this motion-corrected sequence was set up in collaboration with GE applications specialists.

### Image preprocessing

With current multi-channel coils having a higher sensitivity closer to the coils, raw acquired images typically have a higher signal intensity at the surface than in the center of the brain. Corrections performed during image reconstruction can be performed in multiple ways. In the most advanced method, PURE (Phased-array UnifoRmity Enhancement) uses a prescan calibration of the multi-channel coil. The alternative is an image-based method, SCIC (Surface Coil Intensity Correction) filters intensity variations with a low spatial frequency. Given that PURE uses a calibration scan acquired prior to the FLAIR acquisition, reorienting the FOV in PMC scans could lead to a mismatch between calibration and scan coverage, causing a deterioration rather than an improvement in image quality. For this reason, PURE is not compatible with PMC. In consequence, the two groups of subjects had different inhomogeneity corrections, with the non-PMC group having PURE and the PMC group have SCIC correction.

### Image analysis

Visual image quality was scored independently by two experienced radiologists (with 10 and 25 years experience) on six criteria: resolution, SNR, WM-GM contrast, intensity homogeneity, lesion conspicuity, and diagnostic confidence. A seven-point Likert scale (1 = non-diagnostic, 2 = poor, 3 = acceptable, 4 = standard, 5 = above average, 6 = good, 7 = outstanding) was used to rate each criterion (as in [16]). Here, anything below “acceptable” was both classified as being of unacceptable diagnostic quality, with the distinction between “non-diagnostic” and “poor” being that scans in the former category had no useful diagnostic information whereas the latter had some information, but not sufficient to be independently used for diagnosis. The groups of scans (non-PMC, PMC) were randomized into three different batches that were reviewed by both raters in three separate reading sessions with at least one week in between each session.

To complement the visual ratings, SNR was also quantified based on objective measurements of mean and variance of signal intensities. Each subject's 3D T1-weighted image–without PMC, acquired as part of the routine clinical protocol and visually confirmed to be of adequate image quality [17]–was used for a white matter (WM) segmentation using the Geodesic Information Flows algorithm [18]. This probabilistic WM segmentation was thresholded at a probability of 0.95 and eroded by one voxel, after registration to the FLAIR scan. Within this WM mask, 100 voxels were randomly selected, and the 3 × 3 × 3 neighbourhood around each voxel was used to get an SNR estimate (μ/σ over those 27 voxels). The average of those 100 randomly selected SNR samples was taken as that scan's overall SNR.

### Statistical analysis

Statistical evaluation of the visual ratings was performed using the chi-squared test for ordinal data. Here, the expected distribution was assumed to be the ratings for each criterion of the non-PMC scans, and the ratings of the PMC scans were compared to this under the null hypothesis that these are not different. Significance testing using the chi-squared test only checks for significant differences between two sets of histograms, with no conclusion as to which of two groups would have a better image quality. Interpretation of which group had better image quality when a significant difference in the chi-squared test was observed is performed using median and mean values and cumulative histograms of the scores. Here, we define the term “significantly worse” as: a combination of significantly different histograms as indicated by the chi-squared test and a lower median value.

Because median values between groups can be equal, mean values are also reported. Given that the mean of ordinal variables is not necessarily informative (the differences between categories on the Likert scale are not necessarily equidistant) the mean values should be interpreted with care, as for instance through cumulative histograms of the ratings.

Quantitative SNR was compared using Student's t-test over the 40 SNR values for each group to test whether the mean SNR is different between the two groups. Quantitative and visual SNR ratings were compared using correlation analysis, using the Pearson correlation coefficient to utilise the full range of quantitative SNR values.

## Results

There was no evidence for significant differences in age between the two groups (*P* = 0.49 for Student's *t*-test).

**Fig. 1:**
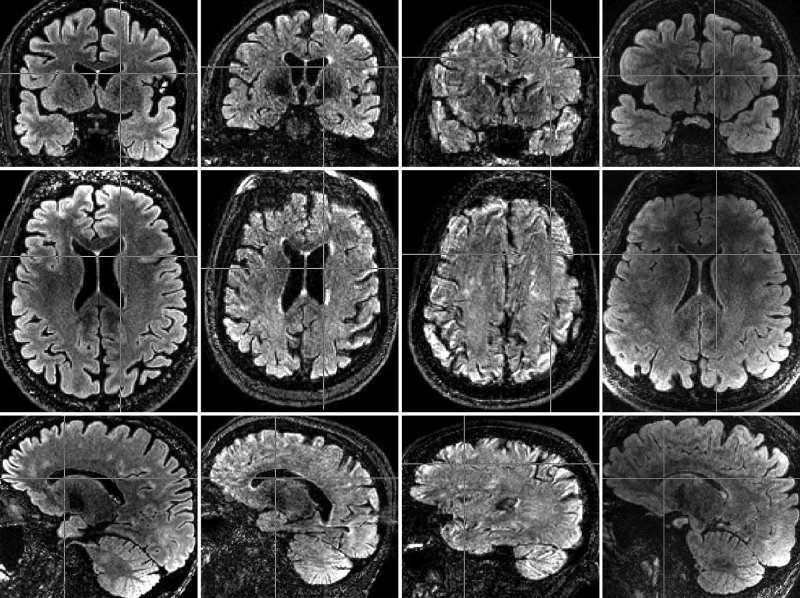
Coronal (top), axial (middle), and sagittal (bottom row) slices of examples of a non-PMC scans with good image quality (left-most column), non-PMC scan with acceptable image quality without obvious motion-corruption (second column) non-PMC scan obviously corrupted by motion (third column), and a PMC scan (right-most column). The two raters score and average score of above-average (5) and standard (4) for the left scan, poor (2) for the second scan, poor (2) and non-diagnostic (1) for the third scan, and standard (4) and average (3) for the right-most scan, respectively.

Fig. 1 shows examples of the normal range of image quality in non-PMC scans, a motion-corrupted non-PMC scan, and a PMC scan in which motion occurred. The PMC scan (Fig. 1, right-most column), had detected motion in 17% of the k-space segments and reacquired these segments for a total extra scan time of 1 m14 s. Even with motion detected in such a large proportion of the segments, image quality is still good despite clear intensity inhomogeneity.

**Fig. 2:**
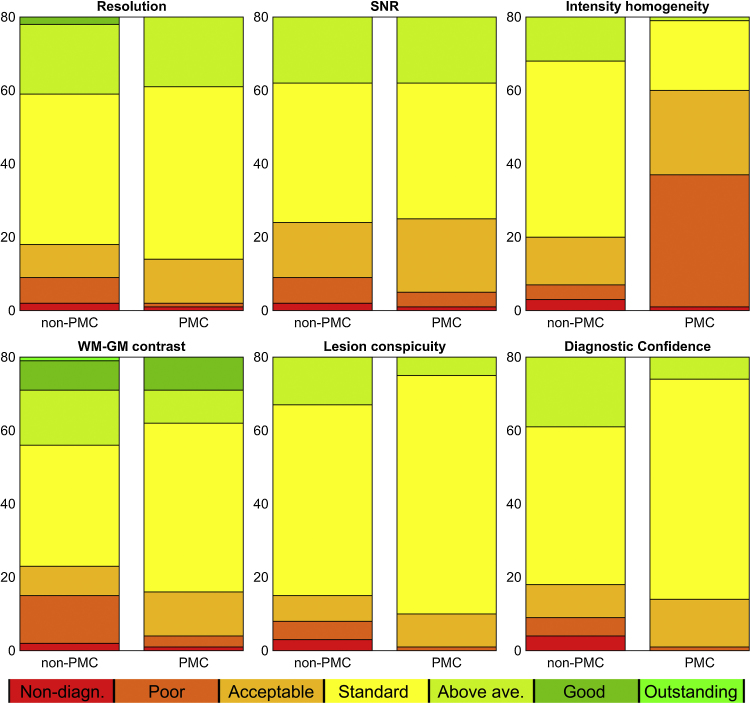
Cumulative histograms of image quality scores for all criteria for the two groups of scans combining the scores from both raters. Color legend is displayed in the bottom of the figure. The use of PMC reduces the number of scans rated non-diagnostic and poor, for all criteria except intensity homogeneity. This comes at the apparent expense of having fewer high-ranking scores (above average and better).

PMC scans had near-identical ratings of image quality to non-PMC scans (Table 2), except that intensity homogeneity was worse on PMC scans, due to the inferior performance of image-based over prescan-based correction. Inspection of cumulative histograms of ratings (Fig. 2) shows lower numbers of low-quality scans in PMC compared to non-PMC, but also lower numbers of highly-rated scans.

**Table 2:** Overall ratings of the scans for all six categories, both scan groups, and both individual raters and a composite score. Reported values are median/mean (standard deviations not reported as they have little meaning on ordinal data).

	Rater 1	Rater 2	Composite
*Resolution*			
Non-PMC	4/3.81	4/4.00	4/3.92
PMC	4/3.91	4/4.09	4/4.00
*SNR*			
Non-PMC	4/3.70	4/3.84	4/3.77
PMC	4/3.79	4/3.84	4/3.82
*Intensity homogeneity*			
Non-PMC	4/3.63	4/3.88	4/3.76
PMC	3/3.23^*^	2/2.35^**^	3/2.80^**^
*WM-GM contrast*			
Non-PMC	4/3.63	4/4.23	4/3.93
PMC	4/3.74^*^	4/4.35	4/4.06^*^
*Lesion Conspicuity*			
Non-PMC	4/3.77	4/3.86	4/3.82
PMC	4/3.86	4/3.95	4/3.92
*Diagnostic Confidence*			
Non-PMC	4/3.72	4/3.93	4/3.83
PMC	4/3.88	4/3.86	4/3.88^**^

Statistical differences in PMC scans are chi-squared tested with respect to non-PMC (*P < 0.01, **P < 0.001, uncorrected). SNR: signal-to-noise ratio; WM: white matter; GM: grey matter; PMC: prospective motion correction.

The main goal of motion-correction is to reduce the number of heavily motion corrupted scans, which is confirmed by a lower percentage of scans with bad image quality (non-diagnostic/poor) with PMC than without PMC on all individual criteria except intensity inhomogeneity (Table 3), for an average of 1.5% vs 8.5% for rater 1 and 5% vs 16.5% for rater 2 – excluding intensity inhomogeneity.

**Table 3:** Percentages of scans with low image quality (non-diagnostic or poor) for each of six criteria. Percentages stated as: average (rater 1/rater 2).

	Non-PMC	PMC
Resolution	11.25 (17.5/5)	5 (5/5)
SNR	11.25 (12.5/10)	6.25 (7.5/5)
Intensity homogeneity	8.75 (15/2.5)	46.25 (20/72.5)
WM-GM contrast	18.75 (25/12.5)	5 (7.5/2.5)
Lesion Conspicuity	10 (12.5/7.5)	1.25 (2.5/0)
Diagnostic Confidence	11.25 (15/7.5)	1.25 (2.5/0)

SNR: signal-to-noise ratio; WM: white matter; GM: grey matter; PMC: prospective motion correction.

Differences between raters was most pronounced in intensity homogeneity and WM-GM contrast. Rater 2 was more critical of the suboptimal inhomogeneity correction in PMC, with 35% of scans having scored two or more points lower than rater [1]. For WM-GM contrast the trend was reversed, with rater 1 scoring two or more points lower than rater 1 in 20% (non-PMC) and 17.5% (PMC). For the other four criteria, mean differences converged to 0 (between −0.25 and 0.25 for each criterion), indicating no clear bias between raters. Mean absolute differences between raters were well below 1 point, indicating a good overall agreement.

**Fig. 3:**
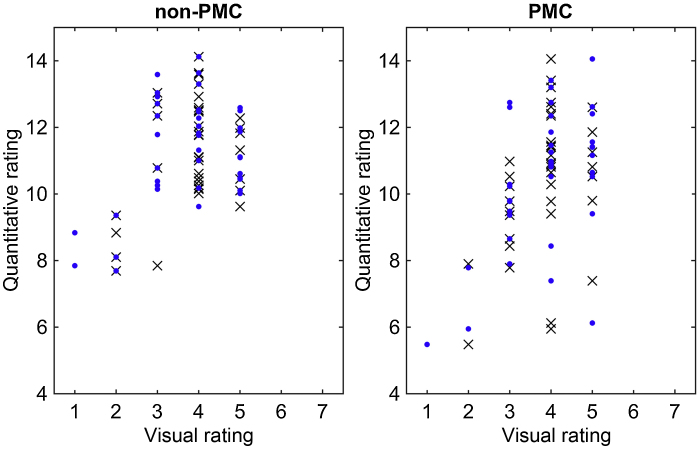
Correlations between visual and quantitative SNR ratings for the two scan groups. The blue dots and black crosses represent the scores from the first and second human rater, respectively.

Quantitative SNR values over the forty scans in each group were (mean ± standard deviation): 11.22 ± 1.59 and 10.46 ± 2.05 for non-PMC and PMC, respectively. Neither groups showed evidence of non-normality (*P* = 0.28 and *P* = 0.21, respectively, on the Lilliefors test for a normal distribution). Student's *t*-test revealed a no significant difference in mean SNR between groups (*P* = 0.07). The correlations between visual and quantitative ratings of SNR is shown in Fig. 3. Pearson correlation analyses for both raters show significant correlations for non-PMC (*P* = 0.009 and *P* = 0.01 for the two raters, respectively) and PMC (*P* < 0.001 and *P* = 0.009) with correlation coefficients between 0.4–0.5. PMC has the highest correlation, which is likely caused by a larger percentage of scans with low SNR in visual scores, effectively increasing the dynamic range. Similarly, the lower correlations in non-PMC could be caused by a rather small dynamic range of values in both visual and quantitative SNR.

The additional scan-time needed for the PMC scans was 17 seconds on average, with 55% of FLAIR scans having no additional scan time and extra scan time once reaching the pre-defined maximum 180 seconds (corresponding to 29 k-space segments being reacquired). In addition to the 7 s increase in scan time in PMC-scans to allow for the navigator readouts, the average rescan time of 17 seconds over the 40 scans increases the total PMC acquisition time to 7 m41 s (compared to the routine acquisition time of 7 m17 s). There was no correlation between additional scan time and diagnostic confidence (*r* = −0.06 and *r* = −0.03 for the two raters). There was no statistical differences in diagnostic confidence for scans without any k-space segments reacquired, compared to scans that did have segments repeated (3.91 vs. 3.83, *P* = 0.49; and 3.95 vs. 3.83, *P* = 0.73; for the two raters, respectively).

## Discussion

To the best of our knowledge, our investigation is the first study of image quality with the use of prospective motion correction in an adult population. We demonstrate that image-based prospective motion correction (PMC) on high-resolution 3D-T2-FLAIR sequences achieves the main goals of PMC which are to prevent subject motion from corrupting image quality and improve diagnostic quality19. Fig. 2 and Table 2, Table 3 show that the percentage of low-quality scans was reduced, increasing the clinical reliability of this sequence.

One remaining issue with PMC is that the intensity filter (SCIC) is suboptimal in removing the inhomogeneity caused by the multi-channel head coils. For individuals with epilepsy specifically, there is a strong recommendation to review FLAIR scans in both axial and coronal orientations, to achieve the highest detection rate possible for subtle focal cortical dysplasias [2], [3]. Intensity inhomogeneity hampers reviewing in planes orthogonal to the acquisition plane (coronal in this study). This is also the main cause for the relatively large interrater difference in intensity homogeneity scores (Table 2, Table 3, Fig. 2), as the rater who scored markedly lower in PMC scans reviewed the scans in all three orthogonal planes, while the other rater generally reviewed images only in the coronal acquisition plane. As described in the Methods section, the recommended prescan-based inhomogeneity correction (PURE) is not compatible with PMC due to expected image quality decline when motion occurs. Seeing no correlation between the amount of motion in our PMC scans and the overall image quality, using the image-based correction (SCIC) does not suffer from this same drawback. One possible way around the intensity inhomogeneity issue is the use of an additional correction step post-acquisition, such as N4 [20]. However, there are logistic difficulties with integrating this into the clinical workflow.

The two other criteria showing significant differences between non-PMC and PMC (WM-GM contrast and Diagnostic confidence) had marginally higher average scores for PMC. Fig. 2 shows that this significant difference in histograms as tested by the chi-squared test is more likely to arise from a sharpening of the histogram (the same median and mode but fewer high-scoring and low-scoring scans) than from a higher average score. Difference between the two raters in WM-GM contrast is driven by a larger spread in ratings in rater 1 than rater 2, as seen in Fig. 2.

Of the six image quality criteria, SNR is the most objectively quantifiable. The difference in contrast within the cortical GM as seen on FLAIR scans (as can be appreciated in Fig. 1) compared to T1 scans and throughout the brain makes it difficult to quantify cortical SNR using a WM-GM segmentation derived from T1 data. We have therefore focused on WM SNR, as the relative differences in SNR between scan groups in WM reflects overall SNR changes in the image.

The increase in scan time of PMC, both the default scan time increase and then rescanning time, is 24 seconds, or about 5% of the non-PMC scan time. This leads to an approximately four-fold decrease in scans with low image quality. Clinically, those scans with low quality might have to be reacquired. This could either be a repeat of the high-resolution 3D-T2-FLAIR, with a likelihood that the repeat scan is motion-corrupted as well, or one would have to revert to 2D sequences with thicker slices to reduce the impact of motion. These two options are both suboptimal in terms of diagnostic quality, which means the reduction in low-quality scans in PMC is worth the relatively small increase in average scan time.

There are various available techniques for prospective motion correction, including in-bore cameras, image-based navigators, and active-markers [13], [19], [21], [22]. The strength of PMC, being a navigator-based approach, is that it requires only changes to the MRI sequence software, with no hardware needed within the bore or attached to the patient (as in for instance [21], [22]. For 3D-T2-FLAIR sequences, the inclusion of multiple navigators in the sequence dead time means a very limited increase in scan time (Table 1) at no other cost (as demonstrated in Fig. 2, Table 2).

Clinical evaluation of prospective motion correction techniques in brain MRI has been limited. The number of brain MRI scans degraded by motion to such an extent as to require a repeat acquisition was 14–35% in a pediatric population [23] and 15% for a general adult population [24], although different criteria were used. For this reason, the initial application of motion correction in brain MRI was in a pediatric setting, where it has been shown to increase diagnostic utility [13], [14]. As stated before, our investigation is the first study of image quality with the use of PMC in an adult population, albeit specifically in epilepsy patients. Further, all clinical evaluations to date have focused on 3D T1-weighted imaging [13], [14]. Given the pronounced difference in sequence timings and contrast between 3D-T1 and 3D-T2-FLAIR scans, the effectiveness of PMC could vary between different imaging contrasts. Irrespective of these differences, we confirm initial conclusions from pediatric populations of an increased diagnostic utility in that more scans were diagnostically useful.

### Limitations

A limitation of this study is that the patients are not the same between the two groups (non-PMC, PMC), precluding a direct comparison of image quality and findings in the same patients. It would, however, not be feasible within a clinical setting to scan a single patient with both acquisitions, nor would there be any guarantee that the scans would have an equal amount of motion. This study, selecting 40 consecutive patients in each group, is the most natural approach in evaluating clinical image quality across scan options.

An inherent limitation of radiological studies in general is the subjective nature of visual ratings, which we have tried to mitigate by quantifying SNR in the WM. Related to the subjective scoring is interrater variability. Generalization is difficult given the difference in how the two raters value the different criteria, and how much information from the three orthogonal orientations they included in their ratings.

## Conclusions

We showed that image-based prospective motion correction (PMC) decreases the proportion of low-quality 3D-T2-FLAIR scans, and hence reduces the number of scans that need to be repeated. This benefit comes at no cost for five of six image quality criteria, with only a lower intensity homogeneity on PMC scans as a potential confounder in reviewing reformatted images.

## Disclosure of interest

The authors declare that they have no competing interest.
